# T1 Mapping for Characterization of Intracellular and Extracellular Myocardial Diseases in Heart Failure

**DOI:** 10.1007/s12410-014-9287-8

**Published:** 2014-07-31

**Authors:** Viviana Maestrini, Thomas A. Treibel, Steven K. White, Marianna Fontana, James C. Moon

**Affiliations:** 1 The Heart Hospital Imaging Centre, University College London Hospitals, 16-18 Westmoreland Street, London, W1G 8PH UK; 2 Department of Cardiovascular, Respiratory, Nephrology, Anesthesiology & Geriatric Sciences, Sapienza University, Rome, Italy; 3 Institute of Cardiovascular Science, University College London, London, WC1E 6BT UK

**Keywords:** T1 mapping, Extracellular space, Heart failure, Extracellular volume fraction, Diffuse fibrosis, Intracellular space, Cardiac remodelling, Myocardial intracellular volume, Myocytes, Interstitium

## Abstract

Heart failure (HF) is a major and growing cause of morbidity and mortality. Despite initial successes, there have been few recent therapeutic advances. A better understanding of HF pathophysiology is needed with renewed focus on the myocardium itself. A new imaging technique is now available that holds promise. T1 mapping is a cardiovascular magnetic resonance (CMR) technique for non-invasive myocardial tissue characterization. T1 alters with disease. Pre-contrast (native) T1 changes with a number of processes such as fibrosis, edema and infiltrations. If a post contrast scan is also done, the extracellular volume fraction (ECV) can be measured, a direct measure of the interstitium and its reciprocal, the cell volume. This dichotomy is fundamental — and now measurable promising more targeted therapy and new insights into disease biology.

## Introduction

Heart failure (HF) is a complex clinical syndrome where the ability of the heart to supply physiological perfusion to organs is impaired, either by acquired or inherited disease. The key biomarker for measuring heart failure is the left ventricular ejection fraction (LVEF) — but a low ejection fraction and HF are not synonymous. Common causes are ischemic heart disease, hypertensive heart disease, valvular heart disease, diabetes, myocarditis, cardiomyopathies, tachycardia-induced cardiomyopathy, and systemic diseases with cardiac involvement. In most HF presentations there is more than one cause. HF represents one of the most important problems for both modern medicine and society with massive impact on individuals and a major annual cost for health systems. The annual costs for HF care exceed $40 billions in the US, with the majority spent on hospitalizations for HF [1–3]. Moreover, hospital discharges for HF remained essentially unchanged over 10 years [4].

Damage the heart and the whole organism responds — upregulated renin-angiotensin system changes, haemodynamic and skeletal muscle changes are the most well known. However, it is the myocardial response that is the most fundamental — that of the myocytes and fibroblast (the most prevalent cells in the myocardium), and extracellular space changes. These changes may be initially adaptive but may progress to be maladaptive. Common features of this remodelling process are cardiomyocyte hypertrophy, extracellular matrix expansion and composition alteration. Calcium handling, energy metabolism, contractile function, vasculature and cell viability may also change. These processes although deeply interwoven may combine into characteristic myocardial phenotypes which, if we can measure them, may permit us to split HF up in a subtypes, opening the door for tailored therapy according to the precise myocardial phenotype [5].

There is however a problem. There is no denying the significant advances in treatment strategies for HF, including drug and device therapy, cardiac transplantation, and mechanical circulatory support. However, despite these, the prognosis has not improved in worsening chronic HF, de novo HF and advanced or end-stage HF, which continue to have high mortality and re-admission rates [2–6]. Whilst the basic biology has pointed to a host of potential therapeutic targets with successful phase 2 trials, few of these have translated to phase 3 trial successes. There may be several reasons. Firstly, we measure the wrong thing — the ejection fraction in defining HF, despite the fact that 40 % of HF has a preserved LVEF. Secondly, we group all types of HF together, thereby ignoring personalised differences in myocardial disease biology. Thirdly, our early phase trial surrogate endpoints (such as short term symptoms improving) are not well tied to important outcomes such as mortality [3]. What is needed is a better practical understanding and measurement of disease biology — fibrosis and myocyte response to split heart failure into different groups, with these biomarkers being tied to specific therapies [2, 3, 7]. This review focuses on a novel technique using standard cardiovascular magnetic (CMR) scanner, T1 mapping, to do this and its potential to act as a disease (rather than syndrome) biomarker, as a surrogate endpoint in trials and as a way of monitoring and tailoring therapy once implemented for better patient outcomes.

## Cardiovascular Magnetic Resonance — LGE Technique

In the last 10 years, CMR has provided two key technologies for HF: anatomical and functional assessment using cine imaging and tissue characterization for focal abnormalities, particularly scar imaging using the late gadolinium enhancement technique (LGE). Cine imaging provided a more accurate and reproducible quantification of LVEF, size and mass, particularly in hearts that were geometrically distorted. The LGE technique for the first time allowed visualization of focal scar. When integrated, there was a step change, but perhaps not a revolution in our understanding of HF. Scar pattern, particularly in early disease provided insights into the underlying causes of myopathy, whilst the extent of scar was prognostic — and incrementally so over LVEF. The lack of scar in particular predicted functional recovery following intervention with resynchronisation (discoordination), revascularization (hibernation) or with time (stunning). Additional techniques such as T2-weighted imaging for edema and T2* imaging for iron quantification added in selected circumstances. However, a gap still remained: the LGE technique could not detect global myocardial changes such as occur in diffuse fibrosis. In addition, all scars looked the same — whether there was 50 % or 100 % myocyte loss in a region. It is in this gap that T1 mapping provides new information.

## T1 Relaxation time and LGE Imaging

Magnetic resonance of protons varies between tissues, depending on the macromolecular environment that water finds itself in. The T1 relaxation time (longitudinal relaxation time, measured in milliseconds) is a tissue-specific magnetic property and is determined by how rapidly protons re-equilibrate their spins within their environment after been excited by radiofrequency pulse. T1 varies with measurement technique and MRI field strength. Regional difference in T1 can be visualized by T1-weighted sequences following an intravenous bolus of extracellular contrast, gadolinium, to evaluate myocardial scar or focal fibrosis.

Gadolinium is an extracellular agent not able to enter through intact cell membrane and cleared from the blood pool after minutes. In tissue with damaged or dead cells, these kinetic effects are delayed and there is a higher accumulation of contrast due to ruptured cell membranes allowing gadolinium to passively diffuse into the cellular compartment. Gadolinium changes magnetic properties by shortening T1 of a tissue. If at this point, a T1-weighted inversion recovery sequence is performed, with the inversion time (TI) set manually by the operator to null “normal” remote myocardium, this will appear black and scar tissue white [8].

The LGE technique is now the gold standard test for the detection of scar across the spectrum of cardiac disease. Its assessment is reproducible and scar visualization provides a good indicator of disease etiology whilst scar extent carries prognostic information, which in many diseases is incremental to conventional prognostic markers. In HF, the LGE technique reduces diagnostic dilemmas but raises new ones: for example, it used to be a clinical conundrum that burnt out hypertrophic cardiomyopathy (HCM) could mimic dilated cardiomyopathy. The LGE technique distinguishes these with ease. However, within the HCM spectrum, whilst in early disease, the LGE pattern may point to the underlying etiology (e.g. sarcomeric protein disease, Fabrys etc.) by end stage, all the diseases start to look the same and scarred out. LGE prognostication is incrementally useful: in a large study (n > 1000), LGE was associated with first hospitalizations for HF after CMR, death or both across the range of ejection fractions including preserved EF. Furthermore, even if LVEF was severely decreased, those without LGE appeared to have less risks of hospitalization for heart failure or death [9].

However, the LGE technique has limits. Measuring the extent of LGE is difficult as different techniques produce different results, particularly in non-ischemic cardiomyopathy [10]. The technique is “black and white” — within LGE areas, complete replacement scar looks the same as 50 % myocyte loss; and conversely background “normal” myocardium is all uniformly nulled meaning diffuse remote pathology and fibrosis is entirely overlooked. This latter point means the LGE technique misses global myocardial pathologies — for example LGE is an uncommon finding in pressure or volume overload disease, systemic condition with cardiac involvement, cardio-toxic effect of different agents, diabetes or hypertension cardiac effect even though all are known to have diffuse fibrosis.

Rapid technical innovations in CMR have generated a novel parametric mapping field addressing these shortcomings. These methodologies permit the routine acquisition of quantitative measure of underlying tissue-specific T1 relaxation rather than relative signal intensities.

## T1 Mapping

**Fig. 1:**
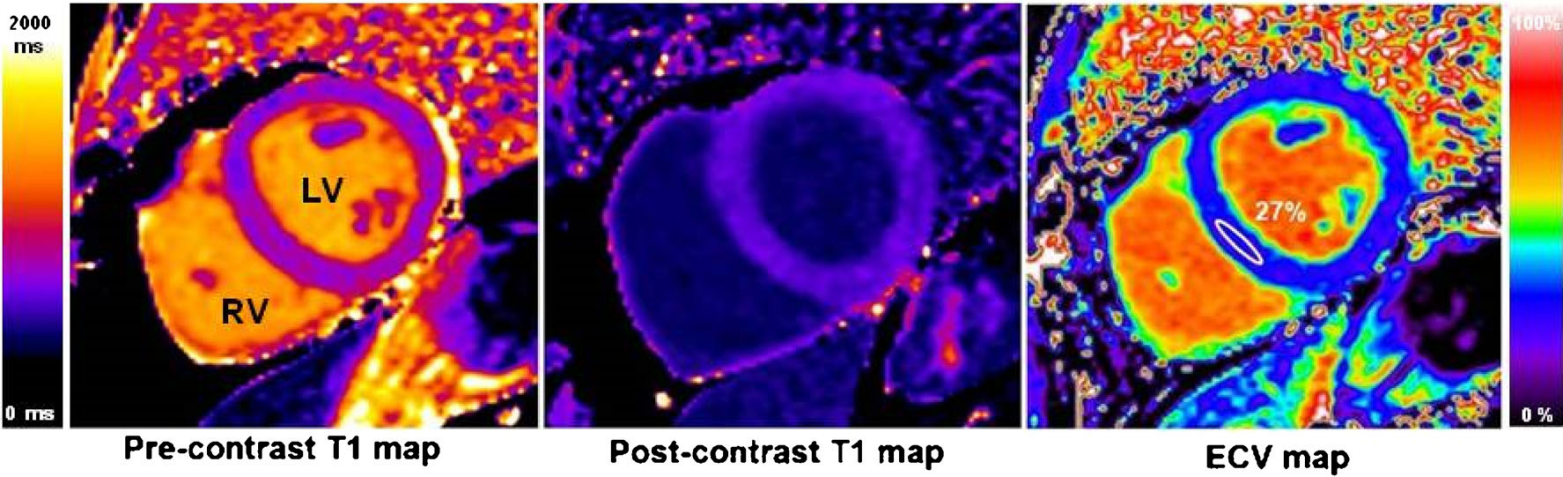
T1 maps (using MOLLI [39•], short axis view) in a healthy volunteer pre-contrast (left) and post-contrast (centre), both measured in milliseconds. For the ECV map (right), each pixel has a value of the interstitial volume as it was calculated from the two T1 maps. The region of interest (white) showing a normal ECV of 27 %

After recent technical improvements, T1 measurement (multi-breath-hold or multiple images requiring curve fitting and processing) has been replaced by T1 mapping. In a single breath-hold, using various approaches, a T1 colour relaxation map is made [11–13]. Within the map, each given pixel value directly corresponds its underlying relaxation time that can be seen (in colour) or more formally measured, standardized, calibrated to histology [14••, 15, 16], compared across diseases and with normal reference ranges [17]. There are two key ways of using T1 mapping: without or before contrast (native T1 mapping); and with contrast, typically by subtracting the pre and post maps with hematocrit correction to generate the extracellular volume fraction (ECV) (Fig. 1).

## Native T1

**Fig. 2:**
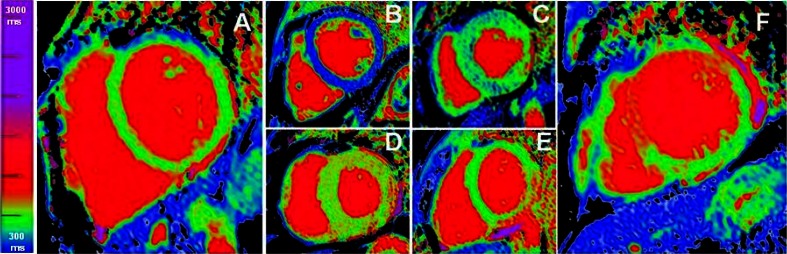
Native T1 maps (using ShMOLLI [12]), all with the same colour scale. (a) healthy volunteer: the myocardium appears homogenously green and the blood is red; low T1 values (blue) from iron overload (c) and lipid storage in Fabry’s disease (c) (except the infero-lateral wall which is high); (d) and (e) represent with high T1 values (red) in amyloid (d) and in myocarditis (d); infarcted (acute infarction) area appears red f) – here basal anter-septum

Myocardial native (non-contrast) T1 measures the T1 values from the extracellular and intracellular compartments. Measurement requires no exogenous contrast administration, making it feasible even in patients with severe kidney dysfunction. T1 *increases* with pathologies where increased water is present such as edema [18, 19] (Fig. 2e), focal or diffuse fibrosis [16] (Fig. 2f) and amyloidosis [20]. T1 *reduces* in the presence of lipid [21] and iron overload (Fig. 2b) [22, 23]. Using a short, single breath-hold, T1 mapping sequence to obtain native T1 values may complement in diseases where the LGE technique works (such as infarction) [18, 19, 24, 25] but more importantly may detect pathology otherwise missed by LGE technique, such as a pan-myocarditis [26]. In diffuse fibrosis, native T1 changes can be subtle. In other diseases, the changes may be very large indeed — two exemplar diseases with gross but opposite T1 changes are informative.

In Anderson-Fabry disease (AFD), where mutations of the α-galactosidase gene result in intracellular lipid accumulation and left ventricular hypertrophy (LVH), the native T1 falls, and falls by many standard deviations (Fig. 2c). This fall appears to be a direct measurement of myocyte lipid storage, which had not previously been measurable. In every other cause of LVH so far explored (HCM, amyloid, aortic stenosis (AS), hypertension) T1 increases, meaning that a low T1 absolutely distinguishes AFD from all other causes of LVH with no apparent overlap [21], and with superior discrimination to other factors [27]. In AFD patients without LVH, up to half of patients have a low T1, suggesting that T1 is a marker of early cardiac involvement. One caveat of interest is that these findings are in the septum in AFD patients without heart failure. In the basal infero-lateral wall a large number of patients have an area of high T1 — in the area that LGE is found, with a surrounding area of normal T1, suggesting a four step pathological progression from normal to low to pseudo-normalized to high T1. Whether this is true (perhaps the infero-lateral wall is never low) and whether this is a pan-myocardial process leading to heart failure is currently unknown. Combined, these observations raise the possibility of T1 mapping for early diagnosis, as a surrogate endpoint in therapy trials and to monitor therapy, but much more work is needed.

In cardiac AL amyloidosis (Fig. 2d), clinically observed cardiac involvement is associated with marked elevations in native T1. These correlate with markers of LV mass, LV systolic dysfunction and markers of diastolic dysfunction (E/E’ and E deceleration time). Clinically, AL amyloid cardiac involvement is classified by echocardiography and biomarkers into absent, possible and definite involvement. T1 is elevated not only in the definite cardiac involvement patients, but also in the possible and no cardiac involvement patients, at a lower level, suggesting both that T1 measurement may add value, but also that amyloid infiltration is an earlier phenomenon in this systemic disease than was previously thought [20]. The other main type of ventricular myocardial amyloid is associated with transthyretin amyloid (TTR; ATTR for amyloid from TTR). In ATTR, preliminary data detect T1 elevations also. These elevations are far higher than fibrotic diseases such as in AS, but appear not as high as in AL amyloid [28].

Native T1 is a feasible clinical tool. However, there are some specific hurdles to be overcome for clinical utility. Whilst pathology influences T1, so does magnet field strength and the precise approach used to its estimation [29]. These differences between approaches may be higher than the differences between health and disease pathology. Therefore at this time whilst technical developments are progressing at pace, normal reference ranges are needed for each approach and ideally for every centre. Moreover, the signal acquired is a composite signal — generated by both interstitium and myocytes. The use of an extracellular contrast agent adds another dimension to T1 mapping and the ability to distinguish and quantify intracellular and extracellular compartment.

## Extracellular Volume Fraction (ECV)

Measuring the T1 time, following the administration of an extracellular contrast agent (gadolinium chelates), generates the possibility to dichotomize the myocardium into its cellular and extra-cellular components. In early studies, the absolute value of T1 post-contrast was used [30] but this has some limitations. This value is affected by renal clearance, gadolinium dose, body composition, acquisition time post bolus and hematocrit. Measuring the ratio of T1 changes pre and post contrast administration in the myocardium and blood provides the partition coefficient, if a sufficient equilibrium of contrast was reached between blood and myocardium [31]. When corrected by hematocrit the myocardial extracellular volume (ECV) is derived [14], biologically representing the myocardial space fraction between cells; more specifically, the space between all cells, including interstitial fluid and plasma between red cells in the myocardial capillaries. Three basic methods have been used: a primed continuous infusion of contrast to reach a definitive equilibrium of distribution between plasma and interstitium; a bolus only approach with sufficient time to elapse post bolus for sufficient equilibrium; and serial time point to curve fit. Currently, it appears that the bolus only approach offers the simplest approach, but further work is needed to clarify whether any incremental benefit of the other two approaches is of sufficient merit [32, 33].

Expansion of the myocardial ECV represents a nonspecific increase in free space between cells and occurs in a variety of pathologies (Table 1). To distinguish, the degree of ECV change and the clinical context is important. Cardiac AL amyloid has a higher ECV than any other diseases generating diagnostic specificity above a certain threshold and seems to detect cardiac involvement earlier than other current tests — and better than native T1 [34]. In absence of amyloid, the increased ECV value expansion is mainly due to edema or increased myocardial collagen. Edema has been little studied to date. ECV imaging can quantitatively characterize infarcted scar and atypical fibrosis, diffuse myocardial abnormalities — even when not clinically apparent on LGE images — and also the small changes occurring in myocardium with aging, even if near to detection limit [35••, 36].

**Table 1:** T1 mapping, ECV and cardiac diseases. Summary of T1 values and ECV for different cardiac diseases

Cardiac disease	Native T1 (msec)	ECV (%)
Healthy volunteer	↔	↔
Severe AS	↑	↑
Chronic MI-LGE	↑↑	↑↑↑
Chronic MI-remote	?	?
Myocarditis	↑↑↑	↑↑**
Amyloid	↑↑	↑↑
HCM-LGE	↑↑	↑↑
HCM-remote	↑	
AFD	↓↓	↔*
Iron overload	↓↓↓	?

Legend: ↔ normal or↑increased or↓decreased T1/ECV; ? (as yet) not known

*data from the septum of AFD patients without heart failure

**data currently only in abstract form

**Fig. 3:**
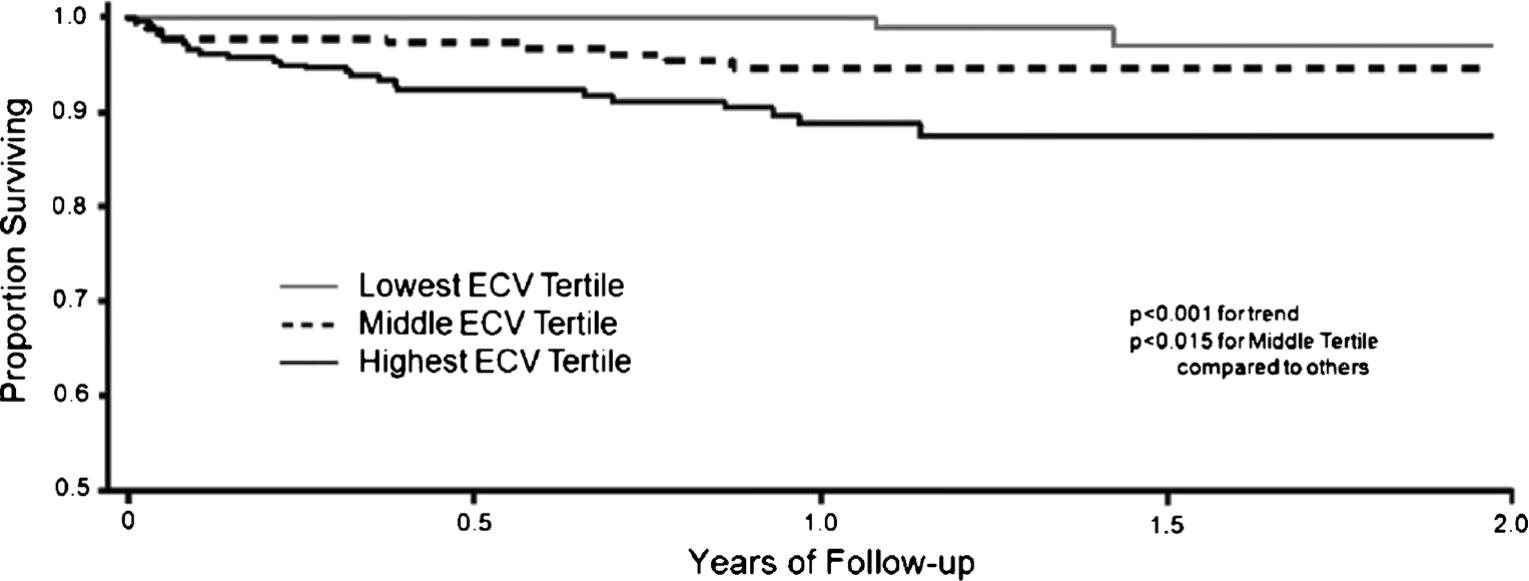
The ECV in non-scar areas (LGE excluded) is associated with all-cause mortality even after relatively short follow-up in all-comers to a CMR service[37••]

For low ECV expansion diseases, biases from blood pool partial volume errors need to be meticulously addressed. Nevertheless, even modest ECV changes appear prognostic. In 793 consecutive patients (excluding amyloid and HCM, measuring outside LGE areas) followed over 1 year, global ECV predicted short term-mortality (Fig. 3) [37••]. The same group also found (n ~ 1000) higher ECVs in diabetics associated with adverse outcome, including mortality and heart failure hospitalization. Those on renin-angiotensin-aldosterone system blockade had lower ECVs. ECV also predicted mortality and/or incident hospitalization for HF in diabetics [38•].

**Fig. 4:**
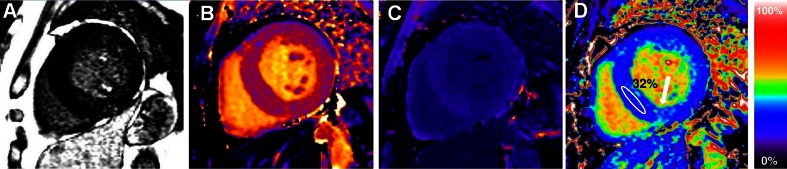
A severe aortic stenosis patient with moderate concentric LVH and patchy scar on LGE imaging (a). Pre-contrast (b) and post-contrast T1 maps (c) and derived ECV map (d) add information: RV insertion point native T1 elevation is seen (b) and there is diffuse extracellular expansion, with an ECV of 32 %

The continuous technical implementation has generated automated parametric ECV maps, now available directly on the scanner, where each pixel carries directly the ECV value improving clinical utility and increasing the possibility to integrate them in clinical protocol (Fig. 4) [39•].

T1 mapping is an exciting and novel tool and transition from early development work to standardized methodologies is crucial. Differences between vendors, sequences, field strength and methodology generate confusion but also innovation, so the early standardization steps are in the form of consensus statements rather than guidelines and emphazise areas where research is needed more so than approaches not to pursue [40••].

## Myocardial Intracellular Volume (ICV)

(1-ECV) represents the myocardium not accessible to the extracellular volume fraction. Accordingly, ICV represents intact myocardial cellular component proving a way to measure the cell volume. Again it is necessary to clarify that there is a bias because, even if ICV mainly represent myocytes, it also includes fibroblasts, blood cells, macrophage, etc.

ICV provides incremental information. In severe AS patients ECV has been found to be elevated but interestingly LV hypertrophy regression after valvular replacement at 6 months had no change in the ECV, but the ICV fell showing non-invasively that early LVH regression was cellular rather than fibrosis regression [41].

For HF, the above approaches are only now starting to be applied. The ability to measure diffuse fibrosis and partition the myocardium into cellular and extracellular compartments is promising. Specifically, it may allow to personalise therapeutic approaches and to develop new therapies targeted either to interstitial or intracellular pathways, but which may otherwise fail if early development applies them indiscriminately to an uncharacterised HF cohort. A number of studies are planned or on their way currently.

## Conclusions

Drug development strategies for HF have produced few positive results over the last decade and HF continues to represent an important problem in medicine with considerable impact for health system cost. To ensure success in mortality reduction a better understanding of disease pathways is needed in-vivo. CMR has established itself as the gold standard for non-invasive myocardial tissue characterization and T1 mapping takes the technology a step further, firstly, by measuring key processes in rare diseases (iron, fat, amyloid), and secondly, by measuring diffuse fibrosis, allowing us to dichotomize the myocardium into its cellular and extra-cellular components, providing new frontiers for pathologies understanding and identifying two different therapeutic targets, cells and interstitium, with high potential impact into our understanding of HF.
